# A deep learning nomogram kit for predicting metastatic lymph nodes in rectal cancer

**DOI:** 10.1002/cam4.3490

**Published:** 2020-09-30

**Authors:** Lei Ding, Guangwei Liu, Xianxiang Zhang, Shanglong Liu, Shuai Li, Zhengdong Zhang, Yuting Guo, Yun Lu

**Affiliations:** ^1^ Department of Epidemiology and Health Statistics The Affiliated Hospital of Qingdao University Qingdao Shandong China; ^2^ Department of Quality Management and Evaluation The Affiliated Hospital of Qingdao University Qingdao Shandong China; ^3^ Shandong Key Laboratory of Digital Medicine and Computer Assisted Surgery Qingdao University Qingdao Shandong China; ^4^ Department of Outpatient Administration The Affiliated Hospital of Qingdao University Qingdao Shandong China; ^5^ Department of Gastrointestinal Surgery The Affiliated Hospital of Qingdao University Qingdao Shandong China; ^6^ State Key Laboratory of Virtual Reality Technology and Systems Beihang University Beijing China

**Keywords:** deep learning, faster region‐based convolutional neural network, lymph node, metastasis, nomogram, rectal cancer

## Abstract

**Background:**

Preoperative diagnoses of metastatic lymph nodes (LNs) by the most advanced deep learning technology of Faster Region‐based Convolutional Neural Network (Faster R‐CNN) have not yet been reported.

**Materials and Methods:**

In total, 545 patients with pathologically confirmed rectal cancer between January 2016 and March 2019 were included and were randomly allocated with a split ratio of 2:1 to the training and validation sets, respectively. The MRI images for metastatic LNs were evaluated by Faster R‐CNN. Multivariate regression analyses were used to develop the predictive models. Faster R‐CNN nomograms were constructed based on the multivariate analyses in the training sets and were validated in the validation sets.

**Results:**

The Faster R‐CNN nomogram for predicting metastatic LN status contained predictors of age, metastatic LNs by Faster R‐CNN and differentiation degrees of tumors, with areas under the curves (AUCs) of 0.862 (95% CI: 0.816‐0.909) and 0.920 (95% CI: 0.876‐0.964) in the training and validation sets, respectively. The Faster R‐CNN nomogram for predicting LN metastasis degree contained predictors of metastatic LNs by Faster R‐CNN and differentiation degrees of tumors, with AUCs of 0.859 (95% CI: 0.804‐0.913) and 0.886 (95% CI: 0.822‐0.950) in the training and validation sets, respectively. Calibration plots and decision curve analyses demonstrated good calibrations and clinical utilities. The two nomograms were used jointly as a kit for predicting metastatic LNs.

**Conclusion:**

The Faster R‐CNN nomogram kit exhibits excellent performance in discrimination, calibration, and clinical utility and is convenient and reliable for predicting metastatic LNs preoperatively.

Clinical trial registration: ChiCTR‐DDD‐17013842.

## Graphical abstract

The deep learning nomogram for predicting metastatic LN status exhibits excellent discrimination of around 0.900, and good calibration and clinical utility. The deep learning nomogram for predicting LN metastasis degree also exhibits excellent discrimination of around 0.900, and calibration and clinical utility. The 2 nomograms can be jointly used as a kit for the preoperative risk prediction of various LN metastases in rectal cancer.

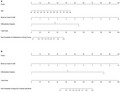

## 1 INTRODUCTION

Rectal cancer constitutes a large part of gastrointestinal tumors, and the mortality rate of colorectal cancer remains high despite its decline over the last 2 decades.^1^, ^2^, ^3^ Among the metastatic pathways of rectal cancer, lymph node (LN) metastasis is the most important, as it usually leads to a poor prognosis due to a high rate of local recurrence.^4^, ^5^, ^6^ Moreover, preoperative evaluation of metastatic LNs is critical for determining the optimal treatment strategies for rectal cancer patients.^7^, ^8^ Magnetic resonance imaging (MRI) has been widely used in clinical practice for diagnosing metastatic LNs in rectal cancer and is considered superior to computed tomography (CT) for its better soft‐tissue discrimination.^9^, ^10^ However, radiologists usually take considerable time to identify metastatic LNs by observing their shapes, boundaries, and signal intensities.^11^ Moreover, different radiologists often have different conclusions with regard to LN metastasis even on the same MRI image.^12^, ^13^, ^14^, ^15^, ^16^ To improve the performance of preoperative metastatic LN diagnosis in colorectal cancer, different kinds of predictive models have been developed, including gene‐ or serum miRNA‐based models and image‐based models.^17^, ^18^, ^19^ Of image‐based models, radiomics derived from CT images are predominant. Radiomics features with different coefficients are first selected from radiological images of a patient and then, are linearly combined for a radiomics score; this work is time‐consuming. The following questions arise: (1) as MRI is superior to CT in diagnosing metastatic LNs, how are the performances of models derived from MRI images, and (2) whether there are any other image‐based methods for rapid and accurate evaluation of metastatic LNs? During the past decade, deep learning technologies have been deeply developed for image recognition; deep learning auto recognition of images has significantly advanced the identification and marking of interesting areas by methods of automatic sketching and 3D reconstruction. It has been used in diagnoses of solid tumors with regard to the skin, lung, breast, and prostate.^20^, ^21^, ^22^, ^23^, ^24^ However, compared with solid tumors, metastatic LNs are more difficult to recognize due to their large quantities and tiny differences. Of deep learning technologies, the most advanced deep learning technology is Faster Region‐based Convolutional Neural Network (Faster R‐CNN), which was developed by Ross b. Girshick in recent years. Faster R‐CNN integrates feature and proposal extractions, bounding box regression, and classification into a complete network and considerably advances the deep learning performance. In the current study, Faster R‐CNN was applied for diagnosing metastatic LNs in rectal cancer and was incorporated into mathematical nomograms for predicting metastatic LNs preoperatively.

## 2 METHODS

### 2.1 Ethics

The Ethics Committee of the Affiliated Hospital of Qingdao University approved the study. The patients gave informed consent. The registration number of the study is ChiCTR‐DDD‐17013842. Patients did not participate in the design, conduct, reporting, or dissemination plans of the study.

### 2.2 Patients

Patients with pathologically confirmed rectal cancer and who met the selection criteria between January 2016 and March 2019 were enrolled in the study, and their clinical data were obtained from the electronic medical system. The inclusion criteria were patients who (1) had pathologically confirmed rectal cancer; (2) had complete clinicopathological data; and (3) received preoperative endoscopic biopsies. The exclusion criteria were patients who (1) were diagnosed with other concurrent malignant tumors; (2) had undergone surgeries for other tumors; (3) were diagnosed with advanced rectal cancer and had not undergone surgeries; (4) received preoperative chemotherapies; and (5) received preoperative radiotherapies. In total, 545 patients were included in the study and were randomly allocated with a split ratio of 2:1 to the training and validation sets, respectively; 362 patients composed the training set, and 183 patients composed the validation set.

Clinicopathological data, including age, sex, differentiation degrees of tumors by preoperative pathology, metastatic LNs by MRI, metastatic LNs by postoperative pathology, carcinoembryonic antigen (CEA) levels, and carbohydrate antigen 199 (CA199) levels, were obtained from the electronic medical system. The levels of CEA and CA199 were analyzed by biochemical tests within 7 days before surgery. A CEA level of ≤5 ng/ml was considered normal, and >5 ng/ml was considered abnormal; a CA199 level of ≤37 U/ml was considered normal, and >37 U/ml was considered abnormal.

### 2.3 Training and validation of Faster R‐CNN

The MRI images of every individual patient with rectal cancer were separately recognized for metastatic LNs by Faster R‐CNN. The architecture of Faster R‐CNN includes detectors that consist of a feature extraction network of feature structures of images in ImageNet and region of interest eigenvectors, as well as regional proposal networks (RPNs). MRI images and a location marking dataset in the training database were used during the training process. ImageNet was initialized by the VGG16 model, and transfer training was performed. The weight initialization of the regional generation networks and region of interest eigenvectors shows a zero mean Gaussian distribution with a 0.01 deviation for the initial random weights. Other training parameters of Faster R‐CNN are shown in Table 1. Detectors and regional generation networks perform end‐to‐end training by stochastic gradient descent and back propagation. The loss function values of the RPN and detectors, as well as the total loss function value of Faster R‐CNN, were output at every iteration of training. More details on the training and validation of Faster R‐CNN are shown in our previous study.^25^

**Table 1:** Training parameters of Faster R‐CNN

Parameters	Values
Iteration	80,000
Learning rate	0.001 before 60000 iterations
	0.0001 after 60000 to 80000 iterations
Momentum	0.9
Weight decay	0.0005
Scale of anchor	8,16,32
Aspect ratio of anchor	1:1,2:1

Faster R‐CNN, faster region‐based convolutional neural network.

### 2.4 Statistical analysis

In univariate analyses, normally distributed continuous variables are presented as the mean (standard deviation, SD), and nonnormally distributed continuous variables are expressed as the median (quartile spacing, Q). Group differences were examined by bilateral *t* test for normally distributed data and rank sum test for nonnormally distributed data. Number and percentage (%) were used to express categorical variables, and group differences were examined by chi‐squared tests (Pearson or continuous correction). In multivariate analyses, logistic regression analyses were performed; the risk degrees of predictors are expressed by the odds ratio (OR) and 95% confidence interval (CI). R software was used for statistical analyses. A two‐tailed *p* value of <0.05 indicates a significant difference.

#### 2.4.1 Nomogram construction

Data from the training set were used for nomogram construction. Using LN metastasis as an outcome variable, the training set included 362 patients with rectal cancer; using LN metastasis degree (at stage N1 or N2) as an outcome variable, the training set included 280 rectal cancer patients with metastatic LNs. Univariate analyses and multivariate analyses were separately performed to identify the significant predictors of an outcome variable. A nomogram for predicting the risk probability of an outcome variable was developed on the basis of multivariate analysis.

#### 2.4.2 Nomogram validation

Using LN metastasis as an outcome variable, the validation set included 183 patients with rectal cancer; using LN metastasis degree (at stage N1 or N2) as an outcome variable, the validation set included 153 rectal cancer patients with metastatic LNs. The performance of a nomogram was validated with regard to capabilities of discrimination, calibration and clinical utility. The area under the curve (AUC) of the receiver operating characteristic (ROC) curve were used to verify the discrimination capability of a nomogram. The calibration plot graphically presenting the predictive probabilities and the observations was used to verify the calibration capability of a nomogram based on resamples of 1000 bootstraps.^26^ Decision curve analysis (DCA) was used to verify the clinical utility of a nomogram, considering realistic threshold probabilities.^27^, ^28^

## 3 RESULTS

### 3.1 Risk prediction of metastatic LN status in rectal cancer patients

#### 3.1.1 Baseline characteristics of the rectal cancer patients in the training set (n = 362) and the validation (n = 183) set

In total, 545 patients were included, including 338 males (62.0%) and 207 females (38.0%), with a mean (SD) age of 58.55 (12.57) years ranging from 26 to 86 years. No difference in any characteristic was observed between groups (all *p* > 0.05; see, Table 2).

**Table 2:** Characteristics between the training set and the validation set

Characteristics	Training (n = 362)	Validation (n = 183)	Statistics	*p*
Mean age, y (SD)	57.98 (12.38)	59.70 (12.89)	−1.514^a^	0.131
Male, n (%)	226 (62.4)	112 (61.2)	0.078^b^	0.780
LN metastasis by pathology, n (%)				
Negative	82 (22.7)	30 (16.4)	2.916^b^	0.088
Positive	280 (77.3)	153 (83.6)		
Differentiation degree, n (%)				
High	142 (39.2)	63 (34.4)		
Moderate	184 (50.8)	93 (50.8)	3.177^b^	0.204
Low	36 (10.0)	27 (14.8)		
CEA Level, n (%)				
Normal	251 (69.7)	126 (68.9)	0.013^b^	0.908
Abnormal	111 (30.3)	57 (31.1)		
CA199 Level, n (%)				
Normal	324 (89.5)	163 (89.1)	0.024^b^	0.877
Abnormal	38 (10.5)	20 (10.9)		

N = 545.

Abbreviations: CA199, carbohydrate antigen 199; CEA, carcinoembryonic antigen; Faster R‐CNN, faster region‐based convolutional neural network; LN, lymph node; MRI, magnetic resonance imaging; SD, standard deviation.

^a^ Statistics for Student's *t* test.

^b^ Statistics for Pearson's Chi‐squared test.

#### 3.1.2 Predictors for metastatic LN status‐Univariate analyses

Predictors of age, metastatic LNs by MRI, metastatic LNs by Faster R‐CNN, differentiation degrees of tumors, and CEA levels were considered significant for LN metastasis (all *P*＜0.05; see Table 3)

**Table 3:** Predictors for metastatic LN status in the training set‐univariate analyses

Predictors	LN‐ (n = 82)	LN+ (n = 280)	Statistics	*p*
Mean age, y (SD)	53.06 (12.57)	59.41 (11.97)	−4.179^a^	<0.001
Male, n (%)	56 (68.3)	170 (60.7)	1.553^b^	0.213
Metastatic LNs by MRI, Median (Q)	0 (2)	2 (3)	−8.703^c^	<0.001
Metastatic LNs by Faster R‐CNN, Median (Q)	0 (2)	2 (4)	−8.967^c^	<0.001
Differentiation degree, n (%)				
High	58 (70.7)	84 (30.0)		
Moderate	22 (26.8)	162 (57.9)	44.844^b^	<0.001
Low	2 (2.4)	34 (12.1)		
CEA Level, n (%)				
Normal	66 (80.5)	185 (66.1)	6.200^b^	0.013
Abnormal	16 (19.5)	95 (33.9)		
CA199 Level, n (%)				
Normal	76 (92.7)	248 (88.6)	1.141^b^	0.285
Abnormal	6 (7.3)	32 (11.4)		

N = 362.

Abbreviations: CA199, carbohydrate antigen 199; CEA, carcinoembryonic antigen; Faster R‐CNN, faster region‐based convolutional neural network; LN, lymph node; MRI, magnetic resonance imaging; SD, standard deviation.

^a^ Statistics for Student's *t* test.

^b^ Statistics for Pearson's Chi‐squared test.

^c^ Statistics for Mann‐Whitney *U* test.

#### 3.1.3 Predictors for metastatic LN status‐Multivariate analyses

For identification of metastatic LNs by Faster R‐CNN based on MRI images, potential predictors of metastatic LNs by Faster R‐CNN and metastatic LNs by MRI were separately incorporated with other potential predictors into stepwise multivariate logistic regression analyses. In MRI‐based multivariate analyses, age, metastatic LNs by MRI and differentiation degrees of tumors were considered significant predictors of LN metastasis, with ORs and 95% CIs of 1.046 (1.021 to 1.073), 1.869 (1.551 to 2.324), and 5.961 (3.519 to 10.566), respectively (see, Table 4). The kappa value of 1.194 indicated no multicollinearity among the significant predictors. In Faster R‐CNN‐based multivariate analyses, age, metastatic LNs by Faster R‐CNN and differentiation degrees of tumors were considered significant predictors of LN metastasis, with ORs and 95% CIs of 1.048 (1.022 to 1.075), 1.871 (1.560 to 2.312), and 5.478 (3.186 to 9.832), respectively (see, Table 4). No multicollinearity was found among the significant predictors, with a kappa value of 2.196.

**Table 4:** Predictors for metastatic LN status in all patients of the training set‐multivariate analyses

Predictors	B	SE	OR (95% CI)	*Z* value	*p*
MRI‐based analyses					
Age	0.0451	0.0126	1.046 (1.021‐1.073)	3.589	<0.001
Metastatic LNs by MRI	0.6256	0.1030	1.869 (1.551‐2.324)	6.076	<0.001
Differentiation degrees	1.7853	0.2798	5.961 (3.519‐10.566)	6.380	<0.001
Faster R‐CNN‐based analyses					
Age	0.0467	0.0128	1.048 (1.022‐1.075)	3.640	<0.001
Metastatic LNs by Faster R‐CNN	0.6262	0.1001	1.871 (1.560‐2.312)	6.258	<0.001
Differentiation degrees	1.7007	0.2868	5.478 (3.186‐9.832)	5.931	<0.001

N = 362.

Abbreviations: *B*, regression coefficient; Faster R‐CNN, faster region‐based convolutional neural network; LN, lymph node; MRI, magnetic resonance imaging; *SE*, standard error of regression coefficient.

#### 3.1.4 Development and validation of nomograms for predicting metastatic LN status

**Figure 1:**
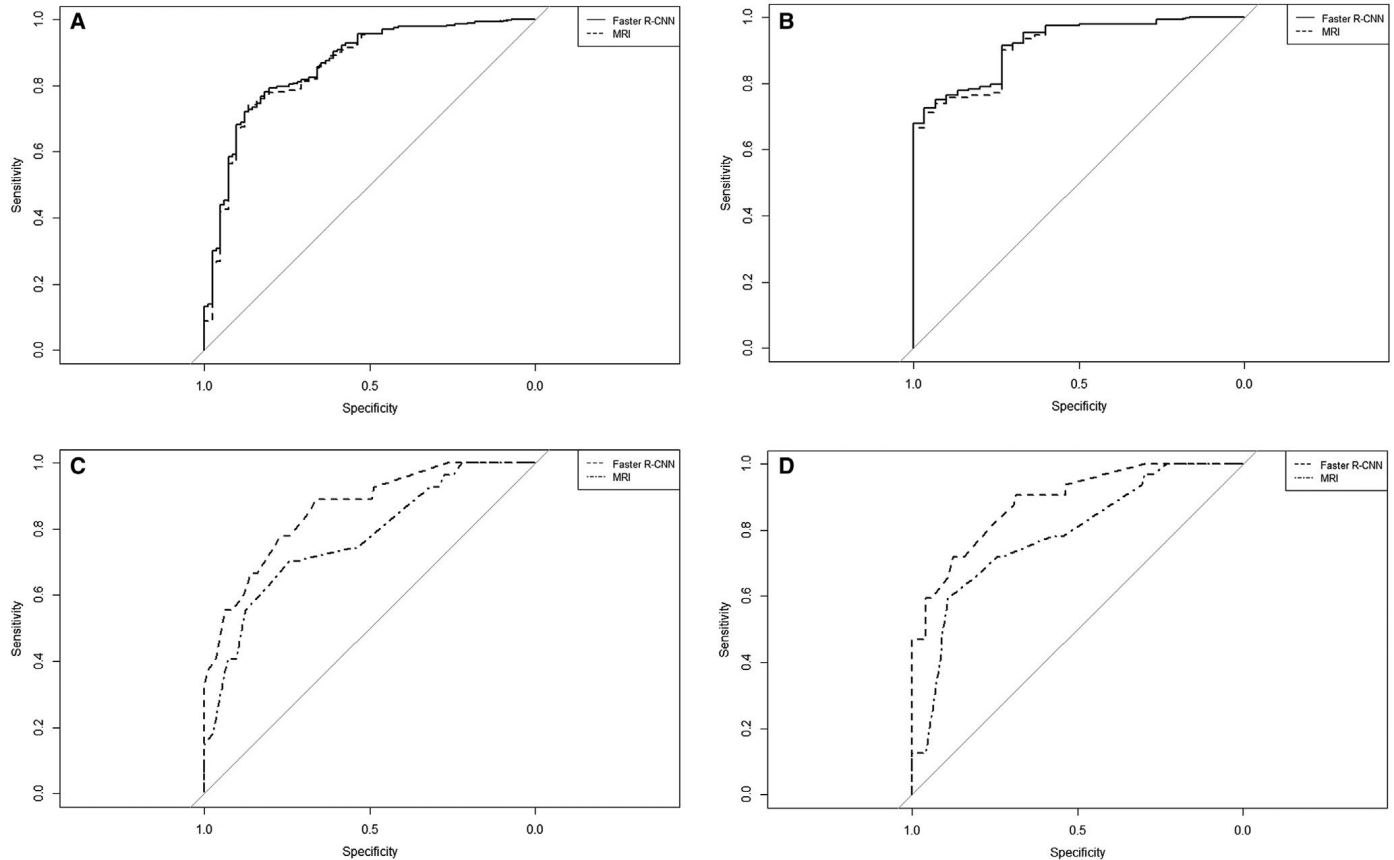
Receiver operating characteristic curves of the nomogram for predicting metastatic LN status in the training set (A), the nomogram for predicting metastatic LN status in the validation set (B), the nomogram for predicting LN metastasis degree (at stage N2 vs N1) in the training set (C), and the nomogram for predicting LN metastasis degree (at stage N2 vs N1) in the validation set (D). LN, lymph node; Faster R‐CNN, faster region‐based convolutional neural network; MRI, magnetic resonance imaging

**Figure 2:**
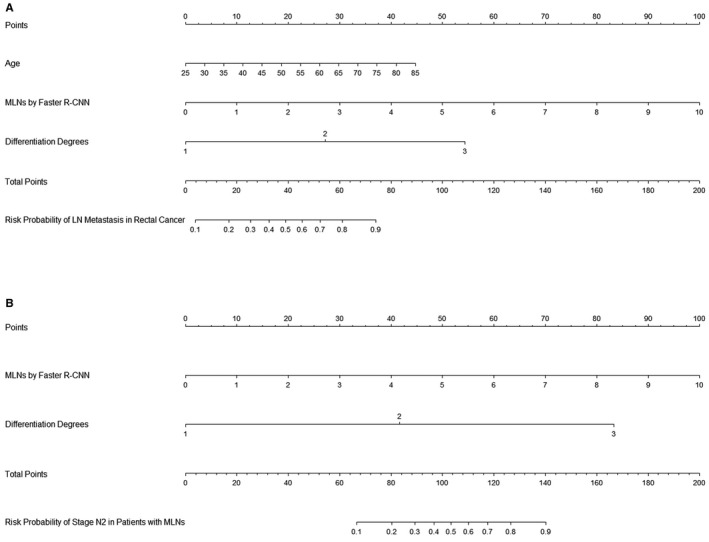
The Faster R‐CNN nomogram for predicting metastatic LN status (A) and for predicting LN metastasis degree (at stage N2 vs N1) (B). Faster R‐CNN, faster region‐based convolutional neural network; MLNs, metastatic lymph nodes; LN, lymph node; Differentiation Degrees: 1 = “well differentiated,” 2 = “moderately differentiated,” and 3 = “poorly differentiated”

Significant predictors identified in MRI‐based and Faster R‐CNN‐based multivariate analyses were separately included for the development of predictive nomograms, and they were the MRI1 nomogram and Faster R‐CNN1 nomogram, respectively. Their AUCs and 95% CIs of 0.856 (0.808‐0.905) and 0.862 (0.816‐0.909), respectively, demonstrated excellent performances in discriminating the risk of LN metastasis in the training set. The two ROC curves were compared by DeLong's test, and a significant difference in the AUC was found between the MRI1 nomogram and Faster R‐CNN1 nomogram (*Z* = −2.652, *p* = 0.008, see, Figure 1A). In the validation set, good performances of the proposed nomograms were also observed, as evidenced by the AUCs and 95% CIs of 0.914 (0.867‐0.960) and 0.920 (0.876‐0.964), respectively. A significant difference in the AUCs was found by DeLong's test between the MRI1 nomogram and Faster R‐CNN1 nomogram (*Z* = −2.179, *p* = 0.029, see, Figure 1B). Therefore, the Faster R‐CNN1 nomogram was considered preferable for predicting metastatic LN status by a linear combination of three predictors (see, Figure 2A).

**Figure 3:**
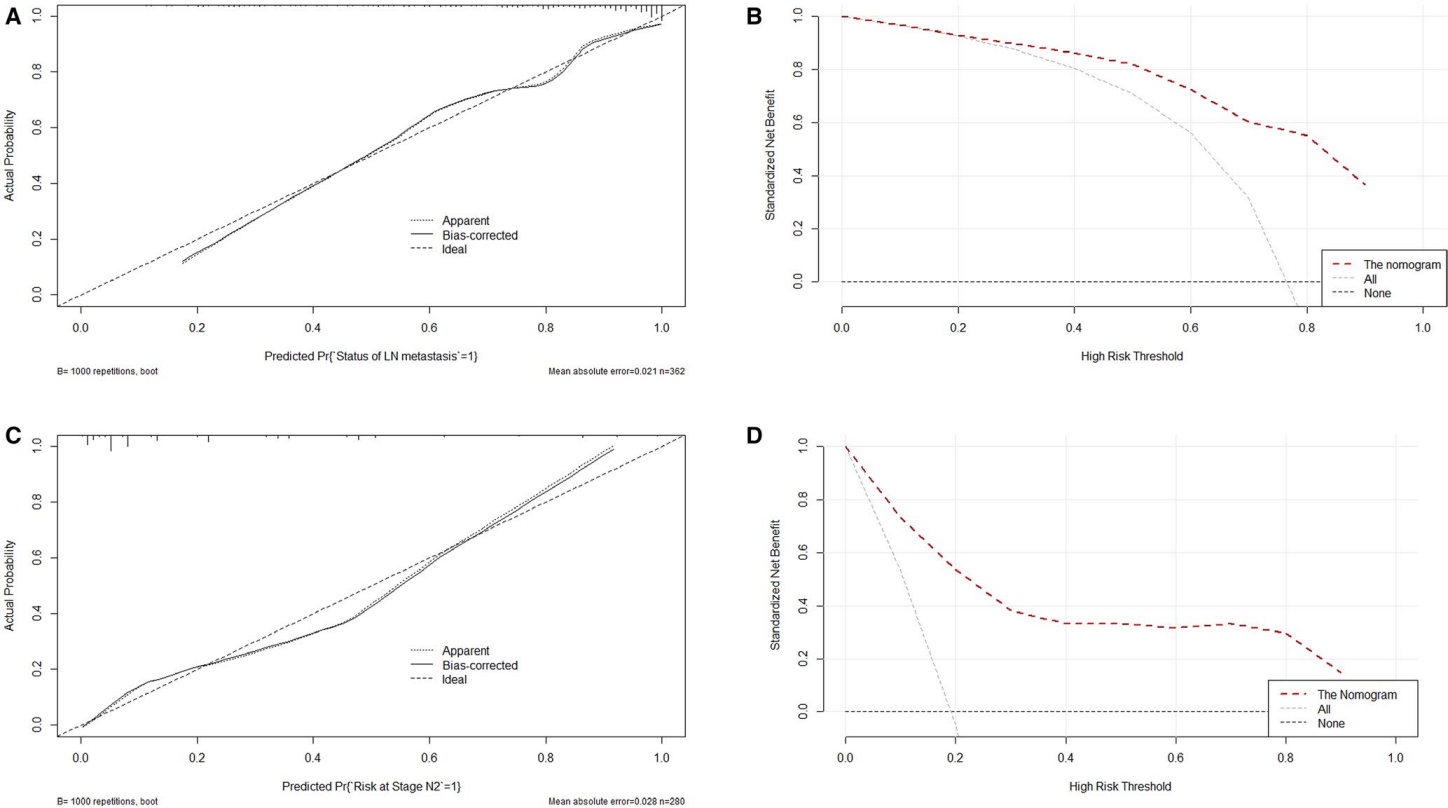
The calibration plot of the Faster R‐CNN nomogram for predicting metastatic LN status (A), decision curve of the Faster R‐CNN nomogram for predicting metastatic LN status (B), calibration plot of the Faster R‐CNN nomogram for predicting LN metastasis degree (at stage N2 vs N1) (C), and decision curve of the Faster R‐CNN nomogram for predicting LN metastasis degree (at stage N2 vs N1) (D). Faster R‐CNN, faster region‐based convolutional neural network; LN, lymph node

The calibration plots demonstrated good consistency between the observations and the predictive probabilities of the Faster R‐CNN1 nomogram (see, Figure 3A). The DCA showed considerable net benefit of the Faster R‐CNN1 nomogram, along with the threshold probabilities (Figure 3B).

### 3.2 The risk prediction of LN metastasis degree (at stage N1 or N2) in rectal cancer patients with metastatic LNs

#### 3.2.1 Baseline characteristics of the rectal cancer patients with metastatic LNs in the training set (n = 280) and the validation (n = 153) set

In total, 433 patients were included in the study, including 260 males (60.0%) and 173 females (40.0%), with a mean (SD) age of 59.86 (12.26) years ranging from 28 to 86 years. No difference in any characteristic was observed between groups (all *p* > 0.05; see, Table 5).

**Table 5:** Characteristics between the training set and the validation set in patients with metastatic LNs

Characteristics	Training (n = 280)	Validation (n = 153)	Statistics	*p*
Mean age, y (SD)	59.41 (11.97)	60.68 (12.77)	−1.027^a^	0.305
Male, n (%)	170 (60.7)	90 (58.8)	0.147^b^	0.701
LN metastasis by pathology, n (%)				
Stage N1	226 (80.7)	121 (79.1)	0.165^b^	0.685
Stage N2	54 (19.3)	32 (20.9)		
Differentiation degree, n (%)				
High	84 (30.0)	45 (29.4)		
Moderate	162 (57.9)	83 (54.2)	1.518^b^	0.468
Low	34 (12.1)	25 (16.3)		
CEA Level, n (%)				
Normal	185 (66.1)	98 (64.1)	0.178^b^	0.673
Abnormal	95 (33.9)	55 (35.9)		
CA199 Level, n (%)				
Normal	248 (88.6)	136 (88.9)	0.010^b^	0.921
Abnormal	32 (11.4)	17 (11.1)		

N = 433.

Abbreviations: CA199, carbohydrate antigen 199; CEA, carcinoembryonic antigen; Faster R‐CNN, faster region‐based convolutional neural network; LN, lymph node; MRI, magnetic resonance imaging; SD, standard deviation.

^a^ Statistics for Student's *t* test.

^b^ Statistics for Pearson's Chi‐squared test.

#### 3.2.2 Predictors for LN metastasis degree in rectal cancer patients with metastatic LNs‐Univariate analyses

Predictors of metastatic LNs by Faster R‐CNN, differentiation degrees of tumors, CEA levels, and CA199 levels were significantly associated with LN metastasis degree (all *p < *0.05; see, Table 6).

**Table 6:** Predictors for LN metastasis degree (N1 or N2) in patients with metastatic LNs in the training set‐univariate analyses

Predictors	Stage N1 (n = 226)	Stage N2 (n = 54)	Statistics	*p*
Mean age, y (SD)	59.32 (11.49)	59.80 (13.90)	−0.261^a^	0.795
Male, n (%)	134 (59.3)	36 (66.7)	0.994^b^	0.319
Metastatic LNs by MRI, Median (Q)	2 (3)	3 (4)	−1.469	0.142
Metastatic LNs by Faster R‐CNN, Median (Q)	2 (3)	5 (2)	−6.255	<0.001
Differentiation degree, n (%)				
High	80 (35.4)	4 (7.4)		
Moderate	132 (58.4)	30 (55.6)	45.588^b^	<0.001
Low	14 (6.2)	20 (37.0)		
CEA Level, n (%)				
Normal	157 (69.5)	28 (51.9)	6.034^b^	0.014
Abnormal	69 (30.5)	26 (48.1)		
CA199 Level, n (%)				
Normal	205 (92.9)	43 (88.9)	5.285^b^	0.022
Abnormal	21 (7.1)	11 (11.1)		

N = 280.

Abbreviations: CA199, carbohydrate antigen 199; CEA, carcinoembryonic antigen; Faster R‐CNN, faster region‐based convolutional neural network; LN, lymph node; MRI, magnetic resonance imaging; SD, standard deviation.

^a^ Statistics for Student's *t* test.

^b^ Statistics for Pearson's Chi‐squared test.

^c^ Statistics for Mann‐Whitney *U* test.

#### 3.2.3 Predictors for LN metastasis degree in rectal cancer patients with metastatic LNs‐Multivariate analyses

Here, potential predictors of metastatic LNs by Faster R‐CNN and metastatic LNs by MRI were also separately incorporated with other potential predictors into stepwise multivariate logistic regression analyses. As shown in Table 7 (MRI‐based multivariate analyses), metastatic LNs by MRI and differentiation degrees of tumors were considered significant predictors of LN metastasis degree in patients with metastatic LNs, with ORs and 95% CIs of 1.338 (1.137‐1.584) and 7.814 (4.175‐15.736), respectively. No multicollinearity was found among the significant predictors, with a kappa value of 1.903. As shown in Table 7 (Faster R‐CNN‐based multivariate analyses), metastatic LNs by Faster R‐CNN and differentiation degrees of tumors were considered significant predictors of LN metastasis degree in patients with metastatic LNs, with ORs and 95% CIs of 1.818 (1.525‐2.218) and 12.077 (5.841‐27.823), respectively. No multicollinearity was found among the significant predictors, with a kappa value of 1.362.

**Table 7:** Predictors for LN metastasis degree (N1 or N2) in patients with metastatic LNs in the training set‐multivariate analyses

Predictors	B	SE	OR (95%CI)	*Z* value	*p*
MRI‐based analyses					
Metastatic LNs by MRI	0.2910	0.0840	1.338 (1.137‐1.584)	3.463	<0.001
Differentiation degrees	2.0559	0.3372	7.814 (4.175‐15.736)	6.098	<0.001
Faster R‐CNN‐based analyses					
Metastatic LNs by Faster R‐CNN	0.5979	0.0949	1.818 (1.525‐2.218)	6.302	<0.001
Differentiation degrees	2.4913	0.3964	12.077 (5.841‐27.823)	6.286	<0.001

N = 280.

Abbreviations: *B*, regression coefficient; Faster R‐CNN, faster region‐based convolutional neural network; LN, lymph node; MRI, magnetic resonance imaging; *SE*, standard error of regression coefficient.

#### 3.2.4 Development and validation of nomograms for predicting LN metastasis degree in rectal cancer patients with metastatic LNs

Significant predictors identified in MRI‐based and Faster R‐CNN‐based multivariate analyses were separately included for the development of predictive nomograms, and they were the MRI1 nomogram and Faster R‐CNN1 nomogram, respectively. Their AUCs and 95% CIs of 0.770 (0.698‐0.842) and 0.859 (0.804‐0.913), respectively, demonstrated good performances in discriminating the risk of stage N2 vs N1 in the training set. A significant AUC difference was found by DeLong's test between the MRI2 nomogram and Faster R‐CNN2 nomogram (*Z* = −3.487, *p* < 0.001, see, Figure 1C). In the validation set, good performances of the proposed nomograms were also observed, as evidenced by the AUCs and 95% CIs of 0.784 (0.694‐0.874) and 0.886 (0.822‐0.950), respectively. A significant difference in the AUCs was found by DeLong's test between the MRI2 nomogram and Faster R‐CNN2 nomogram (*Z* = −3.210, *p* < 0.001, Figure 1D). Therefore, the Faster R‐CNN2 nomogram was considered optimal for predicting LN metastasis degree by calculating two predictors with respective scores (see, Figure 2B).

The calibration plots demonstrated good consistency between the observations and the predictive probabilities of the Faster R‐CNN1 nomogram (see, Figure 3C). The DCA showed considerable net benefit of the Faster R‐CNN1 nomogram, along with the threshold probabilities (Figure 3D).

## 4 DISCUSSION

In this study, the most advanced deep learning technology, that is, Faster R‐CNN, and the mathematical statistical model of the nomogram were merged to predict metastatic LNs preoperatively. A nomogram integrates independent factors of different weights for predicting the risk probability of a clinical outcome visually.^29^ However, Faster R‐CNN nomograms for predicting metastatic LNs have not yet been reported. The study mainly included two aspects of predicting metastatic LNs: the preoperative predictions of metastatic LN status in rectal cancer patients and LN metastasis degree in rectal cancer patients with metastatic LNs.

Predicting metastatic LN status in rectal cancer patients. No significant difference in any characteristic was found between the training (n = 362) and validation (n = 183) sets. The results indicate that selection bias did not impact the results. In univariate analyses for the training set, age, metastatic LNs by MRI, metastatic LNs by Faster R‐CNN, differentiation degrees of tumor, and CEA levels were considered significant for LN metastasis. In the multivariate analyses, predictors of metastatic LNs by MRI and metastatic LNs by Faster R‐CNN were separately incorporated with other potential predictors because of their high correlations with each other. The results showed that age, metastatic LNs by MRI and differentiation degrees of tumors were significant predictors of metastatic LN status in the MRI‐based analyses, and age, metastatic LNs by Faster R‐CNN and differentiation degrees of tumors were significant predictors of metastatic LN status in the Faster R‐CNN‐based analyses. Based on the outcomes of multivariate analyses, the MRI1 nomogram and Faster R‐CNN1 nomogram were separately constructed. In the training set, both of the proposed nomograms demonstrated excellent discrimination capacities, with AUCs of 0.856 and 0.862, respectively. However, the AUC of the Faster R‐CNN1 nomogram was significantly larger than that of the MRI1 nomogram. The results demonstrate that the Faster R‐CNN1 nomogram is preferable and is superior to MRI in diagnosing metastatic LN status. In the validation set, both of the proposed nomograms also demonstrated excellent discrimination capacities, with corresponding AUCs of 0.914 and 0.920, respectively, and the Faster R‐CNN1 nomogram was still considered superior to the MRI1 nomogram. Therefore, the Faster R‐CNN1 nomogram was considered the best model and was graphically presented to predict the risk probability of metastatic LN status by a linear combination of three independent variables with different scores (see, Figure 2A). In addition, the calibration plots demonstrated good consistency between the observations and the predictive probabilities of the Faster R‐CNN1 nomogram. The results confirmed that the nomogram possesses excellent discrimination and calibration. However, a nomogram with excellent discrimination and calibration is not necessarily suitable for clinical application due to its impractical net benefit threshold probabilities.^30^ Therefore, the DCA curves, with threshold probabilities and net benefits on the horizontal and vertical axes, respectively, were plotted to examine the practical value of the nomogram and showed that the Faster R‐CNN1 nomogram yielded clinical benefits. There have been some reported researches using artificial intelligence to predict LN metastasis in rectal cancer. Tse DM developed a computer algorithm by combining morphological features of LNs on MRI to predict LN status in a small sample of 17 patients, and the maximum accuracy was merely 0.86 against radiologists’ diagnoses.^31^ Compared with Faster R‐CNN, its generalization ability and detection effect are much poorer, for most of its learned features are shallow. Moreover, it takes much more time in training. Zhou YP established an automatic recognition system for metastatic LNs of rectal cancer using convolution neural network, and the AUC of the system was 0.89 with reference to radiologists’ diagnoses.^32^ The deep learning technology of this study is on par with ours’, but the incorporation of mathematical nomogram kit into our method makes our prediction on metastatic LNs more accurate, as evidenced by the AUC of 0.92 with reference to pathologists’ diagnoses in the validation set. The Journal of Clinical Oncology reported a CT‐based radiomics nomogram for predicting metastatic LN status in colorectal cancer and showed a relatively good C‐index of 0.736, smaller than the 0.920 of the nomogram proposed by the current study.^19^ In the CT‐based radiomics model, the feature extraction process focuses on the whole tumor outline other than a single metastatic LN as Faster R‐CNN concerns. Some studies reported gene‐related nomograms and miRNA‐related nomograms for the risk prediction of metastatic LN status in colorectal cancer and showed discrimination capabilities of 0.700 and 0.883, respectively.^17^, ^18^ The proposed Faster R‐CNN nomogram is comparable to the gene‐ or miRNA‐related nomogram with regard to the predictive accuracy and even surpasses them. Moreover, all the previous nomograms require plenty of time to extract radiomics features or to conduct gene‐related examinations. However, it takes only 20 s other than the radiologists’ 600 s per case for the Faster R‐CNN to diagnose metastatic LNs.^25^ In addition, all the previous models only predict the risk probability of whether LN metastasis occurs or not; it does not predict the LN metastasis degree (at stage N1 or N2).

Predicting LN metastasis degree (stage N1 or N2) in rectal patients with metastatic LNs. No significant difference was found between the training (n = 280) and validation (n = 153) sets in any characteristic, indicating well‐controlled selection bias. Logistic regression analyses showed that metastatic LNs by MRI and differentiation degrees of tumors were significant predictors of metastatic LN degree in the MRI‐based analyses, and metastatic LNs by Faster R‐CNN and differentiation degrees of tumors were significant predictors in the Faster R‐CNN‐based analyses in rectal cancer patients with metastatic LNs. Then, the MRI2 nomogram and Faster R‐CNN2 nomogram were separately constructed on the basis of the multivariate analyses. In the training set, both of the proposed nomograms demonstrated excellent discrimination capacities, with AUCs of 0.770 and 0.859, respectively; the AUC of the Faster R‐CNN2 nomogram was statistically larger than that of the MRI2 nomogram. The results suggest that the Faster R‐CNN2 nomogram is preferable. In the validation set, both of the proposed nomograms also demonstrated excellent discrimination capacities, with their corresponding AUCs of 0.784 and 0.886, and the Faster R‐CNN2 nomogram was still considered superior to the MRI2 nomogram. The Faster R‐CNN2 nomogram is graphically presented for predicting LN metastasis degree by calculating two independent variables with different scores (see, Figure 2B). In addition, good calibration and clinical utility were confirmed in the nomogram by the calibration plot and the DCA, respectively.

In clinical use, the Faster R‐CNN nomograms for the risk prediction of metastatic LN status and LN metastasis degree should be jointly used as a kit by multiplicative effect methods. For example, a 45‐year‐old man was preoperatively diagnosed with rectal cancer of moderate differentiation by endoscopic biopsy, and the number of metastatic LNs identified by Faster R‐CNN was 6. First, according to the Faster R‐CNN1 nomogram, the total risk score of the patient is calculated to be 135 by sums of the respective points of three predictors, and the corresponding risk probability is considered 1.0 for LN metastasis. Second, the risk probability of metastatic LN degree is calculated to be 0.45 at stage N2 and 0.55 at stage N1 according to the Faster R‐CNN2 nomogram. Therefore, the total risk probability of the patient is 0.45 (1 multiplied by 0.45) at stage N2 and 0.55 (1 multiplied by 0.55) at stage N1. According to the decision curves for the two nomograms, the optimal high risk thresholds are 0.80 for nomogram 1 and 0.25 for nomogram 2, such that the total optimal high risk threshold is 0.20 (0.80 multiplied by 0.25) for stage N2. Therefore, it is preferable to predict the patient at stage N2 for the most clinical benefit.

In addition, there are some limitations to declare. First, the comprehensive performance of Faster R‐CNN and its related nomogram for predicting metastatic LNs are limited to some extent because Faster R‐CNN is based on MRI images other than pathological images. In fact, when the lymph nodes are cut from the body for pathological diagnoses, it is difficult to label each metastatic LN on MRI images. We are looking forward to solving these problems in future studies. Second, the study was performed in one medical group. Though it contains three hospital regions and each region is dozens of kilometers apart, more centers should be involved.

In conclusion, the proposed Faster R‐CNN nomogram kit exhibits excellent comprehensive performance and is convenient and reliable for predicting metastatic LNs preoperatively.

## CONFLICTS OF INTEREST

No conflicts of interest are to be declared.

## AUTHOR CONTRIBUTIONS

Conceptualization: Lei Ding and Yun Lu; Formal analyses: Lei Ding; Data curation: Guangwei Liu, Xianxiang Zhang, and Shanglong Liu; Investigation: Guangwei Liu, Xianxiang Zhang, and Shanglong Liu; Project administration: Lei Ding and Yun Lu; Methodology: Lei Ding, Shuai Li, Zhengdong Zhang, and Yuting Guo; Software: Shuai Li, Zhengdong Zhang, and Yuting Guo; Supervision: Lei Ding and Guangwei Liu; Original draft: Lei Ding; Review and editing: Lei Ding and Yun Lu.

## Data Availability

Data are available upon reasonable request. Individual data that underlie the results reported in this article after de‐identification.
